# Do passive range of motion exercises lead to quicker postsurgical recovery of canine IVDD?

**DOI:** 10.18849/ve.v8i4.670

**Published:** 2023-11-15

**Authors:** Alexander Wallace+

**Keywords:** CANINE, DOGS, INTERVERTEBRAL DISC DISEASE, IVDD, INTERVERTEBRAL DISC EXTRUSION, IVDE, INTERVERTEBRAL DISC HERNIATION, IVDH, PASSIVE RANGE OF MOTION, PROM, RECOVERY TIME, REHABILITATION

## Abstract

**PICO question:**

In canine patients recovering from surgery for intervertebral disc disease, do passive range of motion exercises, compared to no intervention, lead to a shorter or faster rate of recovery?

**Clinical bottom line:**

**Category of research:**

Treatment.

**Number and type of study designs reviewed:**

Five studies (two randomised controlled trials, two retrospective cohort studies and one retrospective case series) were critically appraised.

**Strength of evidence:**

Weak.

**Outcomes reported:**

There is no evidence that passive range of motion (PROM) exercises are associated with a quicker postsurgical recovery in the canine intervertebral disc disease (IVDD) patient.

**Conclusion:**

There is a lack of evidence about specific rehabilitation techniques for the postoperative canine IVDD patient. Based on the current data, a multimodal approach, including basic and intense rehabilitation techniques is suggested.

## How to apply this evidence in practice

The application of evidence into practice should take into account multiple factors, not limited to: individual clinical expertise, patient’s circumstances and owners’ values, country, location or clinic where you work, the individual case in front of you, the availability of therapies and resources.

Knowledge Summaries are a resource to help reinforce or inform decision making. They do not override the responsibility or judgement of the practitioner to do what is best for the animal in their care.

## Clinical scenario

You are presented with a canine patient after they have had decompression surgery for intervertebral disc disease (IVDD) and consider which type of rehabilitation techniques to recommend. An advanced technique may be chosen based on the severity of injury, the equipment available, and owner financial constraints. However, regardless of which, if any, advanced technique is chosen, a basic technique such as passive range of motion exercises should be included in the patient’s rehabilitation plan.

## The evidence

Five studies (two randomised controlled trials, two retrospective cohort studies, and one retrospective case series) were included in this Knowledge Summary. Three studies investigated the effects of basic rehabilitation, including but not limited to passive range of motion (PROM) exercises. However, none of these investigated the effects of PROM exercises alone on recovery. Instead, PROM exercises were one of many basic rehabilitation techniques included in the recovery plan. Two studies compared intense and basic rehabilitation programs and highlighted areas of future investigation. The overall strength of evidence is weak.

## Summary of the evidence

**Bennaim et al. (2017):** 

Population:	Dogs after surgical decompression for Hansen Type 1 intervertebral disc disease (IVDD) with a Modified Frankel Score (MFS) of 3, 4 or 5.
Sample size:	32 dogs.
Intervention details:	32 dogs were randomly split into three groups. Group A (n = 10) received no rehabilitation. Group B (n = 11) received basic rehabilitation. Physical therapy regiment included use of cold packs, passive range of motion exercises, assisted standing exercises, and weight shifting exercises. Group C (n = 11) received basic and intense rehabilitation, in the form of photobiomodulation. Photobiomodulation is the use of low-level lasers to promote tissue repair. Photobiomodulation therapy was administered once a day for five days. All dogs were hospitalised and received rehabilitation therapy for 10 days or until ambulatory. Rehabilitation therapy started 48 hours after surgery, except for cold pack application which was only performed during the first 48 hours. Each dog was assessed with a physical and neurological exam twice per day by the surgeon responsible for the case. Neurologic score and ambulatory status were recorded for 10 days post operation and at last follow-up.
Study design:	Randomised controlled trial.
Outcome studied:	Time to reach recovery grades B, C, and D. Recovery grade B: able to support weight with some help. Recovery grade C: initial voluntary pelvic limb movement. Recovery grade D: ambulatory.
Main Findings (relevant to PICO question)	There was no significant difference (P = 0.26) in recovery time between groups that received rehabilitation and those that did not receive rehabilitation. Time to reach recovery stages B, C, and D did not differ among groups. There was no significant difference in recovery rate between groups.
Limitations:	Inclusion of dogs with and without nociception even though this is a known factor effecting recovery time. Although the presence of dogs without nociception was even among groups. Only a 10 day period of observation took place.

**Ruddle et al. (2006):** 

Population:	Dogs after surgical decompression for Hansen Type 1 intervertebral disc extrusion from two private referral specialist practices between June 2001 and June 2004.
Sample size:	308 dogs.
Intervention details:	Dogs were categorised into two groups. Group A (n = 228) received no rehabilitation. Group B (n = 22) received basic and intense rehabilitation. Including passive range of motion, balance, strength and underwater treadmill training exercises. Rehabilitation was performed by a certified canine rehabilitation practitioner. Dogs were monitored at each rehabilitation session, which was usually 1 hour every week for 6 weeks. Some follow-up examinations were performed as late as 12 weeks post operation. When necessary, this was done over the telephone.
Study design:	Retrospective case series.
Outcome studied:	Return of ambulation. Time to reach ambulation.
Main Findings (relevant to PICO question)	Intense and basic rehabilitation did not significantly influence time to first ambulation. Intense and basic rehabilitation did not significantly influence return of ambulation. Of dogs who did not receive rehabilitation, 90% (205/228) of cases had return of ambulation. Of dogs who received rehabilitation, 77% (17/22) of cases had return of ambulation. The remaining 58 dogs from the original 308 were excluded due to lack of complete data sets and follow up information.
Limitations:	Nature of retrospective case series study with vast amounts of data but short summaries: A summary but no data was provided comparing postoperative physical therapy and time to first ambulation. Lack of tables and figures comparing factors. Of the nine factors studied only the data comparing two, deep pain perception and time to ambulation, were shown.

**Jeong et al. (2019):** 

Population:	Dogs after surgical decompression for Hansen Type 1 intervertebral disc herniation (IDVH) from the Royal Animal Medical Center (Seoul) between 2012 and 2017.
Sample size:	186 dogs.
Intervention details:	Dogs were categorised into two groups. Group A (n = 90) received no rehabilitation. Group B (n = 96) received basic and intense rehabilitation. This program included neuromuscular electrostimulation, infrared treatment, passive standing training, balance board training, deep tendon reflex stimulation, and underwater treadmill training. Neurological assessments were conducted on the day of surgery, 7 days, 14 days, 1 month, and 3 months post operation. Follow-up examinations and telephone interviews took place up to 5 months post operation.
Study design:	Retrospective cohort study.
Outcome studied:	Neurological function before surgery. Preoperative neurological grading system. Grade 2: conscious proprioception (CP) deficits, and paraparesis. Grade 3: CP deficits and weak or non-ambulatory paraparesis. Grade 4: paraplegia with or without deep pain perception. Recovery of neurological functions. Determined by unassisted standing times, unassisted walking times and by Olby score based on neurological examination (Olby et al., 2001).
Main Findings (relevant to PICO question)	The implementation of a rehabilitation program including basic and intense exercises significantly (P < 0.01) increased the recovery rate of dogs after decompression surgery for IVDH. 52% (47/90) of dogs in group A had a neurological recovery. 86% (83/96) of dogs in group B had a neurological recovery. A combination of basic and intense rehabilitation did not significantly reduce time to recovery. Dogs with neurological function grades of 2, 3, and 4 did not have significant difference in time to recovery between those that received rehabilitation and those that did not.
Limitations:	Does not state their own limitations. Does not provide statistical information about the different rehabilitation programs for different patients. The paper only states that they were tailored to each patient based on the animal’s condition.

**Zidan et al. (2018):** 

Population:	Dogs under 20 kg after surgical decompression for thoracolumbar intervertebral disc herniation (IDVH).
Sample size:	30 dogs.
Intervention details:	30 dogs were randomly split into two groups. Group A (n = 15) received basic rehabilitation which included passive range of motion (PROM) exercises and sling walking for 14 days post operation. Group B (n = 15) received intensive rehabilitation which included the basic rehabilitation stated above and supported standing, neuromuscular electrical stimulation, weight shifting exercises, balance board exercises, and underwater therapy for 14 days post On day 15, all dogs from both groups were sent home. Owners were shown how and told to perform PROM and sling walking exercises daily. Owners were contacted once per week to ensure compliance. Dogs had follow-up examinations on days 28 and 42 post operation.
Study design:	Randomised controlled trial.
Outcome studied:	Open field gait score (OFS) ranging from 0–12, at 14, 28, and 42 days. Number of days until independent walking. Treadmill-based coordination score, ranging from 0–100%, at 14, 28, and 42 days.
Main Findings (relevant to PICO question)	There was no significant difference in recovery time and rate between the groups receiving basic and intensive rehabilitation. Median number of days until independent walking was 5 for group A and 8 for group B. This difference was not significant (P = 0.46). Mean change in OFS at day 14 was 6.13 ± 1.26 for group A and 5.73 ± 0.8 for group B. This difference was not significant. A treadmill-based coordination score on day 14 of 55.13 ± 18.25 for group A and 51.65 ± 20.68 for group B. This difference was not significant. Median change in OFS from day 1–42 for group A was 8 (range 3–11) and for group B was 7 (range 5–10). This difference was not significant (P = 0.69).
Limitations:	After 14 days, dogs were released back to owners and owners were instructed to complete basic rehabilitation exercises. However, there is no certainty that these daily exercises were completed. Lack of control group that did not receive basic or intense rehabilitation.

**Hodgson et al. (2017):** 

Population:	Dogs under 20 kg with Hansen Type 1 intervertebral disc herniation (IDVH) that underwent a single site hemilaminectomy from Central Texas Veterinary Specialty Hospital between January 2009 and March 2015.
Sample size:	248 dogs.
Intervention details:	Patients were categorised into two groups. The rehabilitation group (n = 87) received basic and intense rehabilitation. This included land treadmills, underwater treadmills, standing weight shifting exercises, cavaletti drills, sit to stand exercises, cart assisted ambulation, and photobiomodulation therapy. Intensity of the rehabilitation program was tailored to each patient. The control group (n = 161) received basic rehabilitation only. Basic rehabilitation included passive range of motion (PROM), exercises, cryotherapy, and low-level laser therapy. Cryotherapy and PROM exercises were performed for 10 minutes 2–3 times per day. Low-level laser therapy was performed once per day. Dogs received a median of 12 days (range 3–66) of rehabilitation over a median period of 6 weeks (range 1–40).
Study design:	Retrospective cohort study.
Outcome studied:	Time at start of posthospitalisation rehabilitation. Type of posthospitalisation rehabilitation. Time to return of conscious proprioception. Time to ambulation. Presence of complications.
Main Findings (relevant to PICO question)	Basic rehabilitation alone was associated with a faster recovery of neurological signs (P < 0.0001). Median time to ambulation for rehabilitation group was 28 days. Median time to ambulation for control group was 14 days. The combination of intense and basic rehabilitation was associated (P < 0.001) with a higher rate of recovery. Of dogs in the rehabilitation group, 33% (29/87) returned to a modified Frankel score (MFS) of 5. Of dogs in the control group, 9% (8/161) returned to a MFS of 5. Posthospitalisation rehabilitation was associated (P = 0.03) with less postoperative complications. Of dogs in group A, 16% (14/87) had complications. Of dogs in group B, 29% (47/161) had complications.
Limitations:	Dogs with delayed recovery may have begun posthospitalisation rehabilitation as a result. Accuracy of data on in-home rehabilitation completed by owners. Higher prevalence of dogs without deep pain presence in the rehabilitation group.

## Appraisal, application and reflection

### Introduction

The goal of this Knowledge Summary was to investigate if in canine patients recovering from surgery for intervertebral disc disease (IVDD), does the implementation of passive range of motion (PROM) exercises, compared to not implementing PROM exercises, lead to a quicker recovery?

Five studies (two randomised controlled trials, two retrospective cohort studies, and one retrospective case series) were critically appraised to investigate this PICO question. No study was found that directly investigated this PICO question. Two studies (Bennaim et al., 2017; and Ruddle et al., 2006) indirectly investigated this PICO question. One study (Jeong et al., 2019) investigated a similar PICO question but included additional variables. The final two studies (Zidan et al., 2018; and Hodgson et al., 2017) investigated a similar PICO question about the effects of intense rehabilitation therapies. While the lack of directly relevant studies weakens the level of evidence of this Knowledge Summary, it can serve to attract future research.

Basic rehabilitation is defined as PROM exercises, cold pack application, assisted, leash, or ground treadmill walking. Intense rehabilitation types included photobiomodulation, under water treadmill training, and neuromuscular electrostimulation. Additionally, this report defined recovery as when patients were able to walk independently, also known as ambulation. Exclusion criteria included review papers, lack of prior spinal decompression surgery, and inclusion of patients with comorbidities such as diabetes and myelomalacia.

A level of evidence system (Elsevier Author Services) was used to value each critically appraised paper. Level 1 consisted only of randomised controlled trials. Level 2 consisted of non-randomised controlled trials. Level 3 consisted of retrospective cohort studies. Level 4 consisted of case series.

### Effect of basic rehabilitation alone on the postoperative canine IVDD patient

One study (Bennaim et al., 2017) investigated the effect of basic rehabilitation alone on the postoperative canine IVDD patient. They found there was no significant difference in time to ambulation or recovery rate between groups that received basic rehabilitation and groups that did not. They had a level 1 evidentiary value. These findings were consistent with another study that investigated the effects of basic rehabilitation on the non-surgical canine IVDE patient (Jadeson et al.,1961). Bennaim et al. (2017) additionally reported no adverse effects likely due to rehabilitation.

Caveats were identified. No research study was found that investigated PROM exercises alone on the postoperative canine IVDD patient. Rather the study above compared the effects basic rehabilitation programs including but not limited to PROM, cold pack application, balancing and standing exercises. A common caveat of retrospective studies, including three of the five appraised (Ruddle et al., 2006; Jeong et al., 2019; and Hodgson et al., 2017), was that the patients with more severe injuries were more likely to receive intense rehabilitation.

### Additional information on the effect of intense rehabilitation

Two studies (Jeong et al., 2019; and Ruddle et al., 2006) investigated the effect of both basic and intense rehabilitation compared to no rehabilitation on the postoperative canine IVDD patient. Jeong et al. (2019) found a significant difference in recovery rate but not time to recovery. They had a level 3 evidentiary value. Ruddle et al. (2017) found no significant difference in recovery rate or time. They had a level 4 evidentiary value.

Caveats were identified. One was that Jeong et al. (2019) did not isolate PROM exercises. Additionally, this study incorporated passive standing exercise instead of traditional PROM exercises, such as simple flexion and extension by a physiotherapist, which some may not consider PROM exercises.

The inclusion of an additional variable, intense rehabilitation, does not allow us to isolate whether it was the intense or basic rehabilitation that is associated with improved recovery rate. Additionally, this study highlights the effects of intense rehabilitation, such as aquatic treadmill training, on the postoperative canine IVDD patients should be further investigated.

**Table 1: Time to reach ambulation in no rehabilitation group and intense and a basic rehabilitation group (Jeong et al., 2019):** 

Preoperative neurological grade	Group A (No rehabilitation)	Group B (Intense and basic rehabilitation)
Mean number of days until standing from preoperative neurological grade 2	7.0 ± 3.2	6.9 ± 2.2
Mean number of days until standing from preoperative neurological grade 3	7.9 ± 3.2	7.0 ± 2.5
Mean number of days until standing from preoperative neurological grade 4	14.4 ± 3.6	13.0 ± 2.10

Two studies (Zidan et al., 2018; and Hodgson et al., 2017) compared the effect of both intense and basic rehabilitation to basic rehabilitation alone on the postoperative canine IVDD patient. Zidan et al. (2018) found no significance in recovery time or rate. This study had a level 2 evidentiary value and was not consistent with other studies. Hodgson et al. (2017) found a significant improvement in recovery rate but a decrease in time to recovery in those receiving intense and basic rehabilitation compared to basic rehabilitation alone. This study had a level 3 evidentiary value and was consistent with another study of intense rehabilitation (Bruno et al., 2020).

Caveats were identified. First, neither study isolated PROM exercises. Second, in Hodgson et al. (2017) patients with more severe clinical signs were more likely to receive intense rehabilitation.

**Table 2: Summary of critically appraised papers:** 

Study	No rehabilitation group in study	Basic rehabilitation group in study	Intense rehabilitation group in study	Conclusion	Level of evidence
Bennaim et al. (2017)	A	B C	C	Basic or intense rehabiliation made no difference in recovery time or rate	1
Ruddle et al. (2006)	A	B	B	Basic rehabiliatation made no difference in recovery time or rate	4
Jeong et al. (2019)	A	B	B	Intense and basic rehabiliatation helped recovery rate but not time	3
Zidan et al. (2018)	/	A B	B	Intense rehabilitation made no difference in recovery time or rate	2
Hodgson et al. (2017)	/	A B	B	Intense rehabilitation helped recovery rate but decreased time	3

### Conclusion

Of the five papers critically appraised, only two (Bennaim et al., 2017; and Ruddle et al., 2006) investigated the effects of basic rehabilitation, including but not limited to passive range of motion (PROM), exercises, on recovery time and rate. Both papers concluded that there was no significant difference in recovery time or rate between patients that completed PROM exercises and those that did not. The findings of Bennaim et al. (2017) were consistent with another study that investigated the effects of basic rehabilitation on the non-surgical canine intervertebral disc disease, IVDD, patient (Jadeson et al.,1961).

Of the five papers, only Jeong et al. (2019) concluded that PROM exercises in combination with other basic and intense rehabilitation techniques was effective in improving recovery rate. None of the five papers concluded that rehabilitation, intense or basic, improved recovery time.

Unfortunately, there is a lack of research on specific basic rehabilitation techniques for the postoperative canine IVDD patient. Of the papers available, two suggested that basic rehabilitation helps the non-surgical IVDD patient (Jadeson, 1961; and Sikes, 1989). Another paper suggested that only time from onset of clinical signs to surgery and severity of injury affects recovery time. Additionally, this paper suggested that more severely injured patients had a greater chance of recovery through basic rehabilitation (Immekeppel et al., 2021).

Rehabilitation has long been incorporated into human medicine, but only recently has it become common in veterinary medicine. Prior to this, the research on basic rehabilitation had been done primarily on humans and laboratory animals. By the time basic rehabilitation was incorporated into veterinary medicine, researchers were ready to investigate more intense techniques such as photobiomodulation and underwater treadmill training. One study found that electroacupuncture use in the postoperative IVDD patient was associated with a faster recovery time (Hayashi at el., 2007). Other studies have found conflicting results on the effect of photobiomodultion on rate of recovery (Bennaim et al., 2017; and Bruno et al., 2020).

None of the appraised papers reported increased rates of adverse effects. Additionally, Hodgson et al. (2017) reported a significantly (P = 0.03) lower complication rate, 16% (14/87) compared to 29% (47/161), in dogs that received a post-hospitalisation rehabilitation program. Common adverse effects of spinal injury are myelomalacia, a fatal softening of the spinal cord, gastrointestinal distress, and euthanasia due to lack of improvement.

There is contradictory evidence on the effectiveness of PROM exercises, basic rehabilitation, and intense rehabilitation on recovery time for the postoperative canine IVDD. In a 2015 survey, 64% (103/181) of surgeons and 46% (46/105) neurologists routinely recommend some form of rehabilitation. Additionally, neurological status, financial restrictions, and patient body condition were identified as common factors (Moore et al., 2016). Possibly due to a lack of supported rehabilitation techniques, a multimodal approach is commonly chosen. This combination is safe and potentially improves the chance of recovery.

A cross-sectional investigation concluded that in humans with spinal cord injuries, more physical exercise was associated with a higher quality of life (Stevens et al., 2008). If we apply these findings to canine IVDD patients, then providing physical exercise is a necessity for veterinarians.

To summarise, current evidence does not suggest that in patients recovering from surgery for IVDD, PROM exercises lead to a quicker recovery. The strength of evidence is weak as many studies did not isolate and investigate PROM exercises. Instead, they investigated PROM exercises in a combination of other basic or intensive therapies. Additionally, many studies were retrospective. No basic rehabilitation technique has been isolated to affect recovery time. Instead, a multimodal approach to rehabilitation may be important to treating the postoperative canine IVDD patient.

## Methodology

**Search strategy:** 

Databases searched and dates covered:	CAB Abstracts 1961–2023 (Via CAB Direct)(Included products: CAB Abstracts, VetMed Resource, CABI Full Text, and Global Health) PubMed 1961–2023 Google Scholar 1961–2023
Search strategy:	CAB Abstracts: (Canine OR Dog) AND Intervertebral (Disc OR Disk) (Disease OR Herniation) AND Passive Range of Motion OR (Canine OR Dog) AND Intervertebral (Disc OR Disk) (Disease OR Herniation) AND Rehabilitation OR (Canine OR Dog) AND Intervertebral (Disc OR Disk) (Disease OR Herniation) AND Recovery OR (Canine OR Dog) AND Intervertebral (Disc OR Disk) (Disease OR Herniation) AND Outcome PubMed: (Canine OR Dog) AND Intervertebral (Disc OR Disk) (Disease OR Herniation) AND Passive Range of Motion OR (Canine OR Dog) AND Intervertebral (Disc OR Disk) (Disease OR Herniation) AND Rehabilitation OR (Canine OR Dog) AND Intervertebral (Disc OR Disk) (Disease OR Herniation) AND Recovery OR (Canine OR Dog) AND Intervertebral (Disc OR Disk) (Disease OR Herniation) AND Outcome Google Scholar: (Canine OR Dog) AND Intervertebral (Disc OR Disk) (Disease OR Herniation) AND Passive Range of Motion OR (Canine OR Dog) AND Intervertebral (Disc OR Disk) (Disease OR Herniation) AND Rehabilitation OR (Canine OR Dog) AND Intervertebral (Disc OR Disk) (Disease OR Herniation) AND Recovery OR (Canine OR Dog) AND Intervertebral (Disc OR Disk) (Disease OR Herniation) AND Outcome
Dates searches performed:	27 Aug 2023

**Exclusion / inclusion criteria:** 

Exclusion:	Case studies, review papers, laboratory animal and human studies.
Inclusion:	Compared basic, intensive, or no rehabilitation in canine IVDD patients after decompression surgery.

**Search outcome:** 

Database	Number of results	Excluded –Not relevant	Excluded – Review Paper	Total relevant papers
CAB Abstracts				
PubMed				
Google Scholar				
Total relevant papers when duplicates removed				

### ORCiD

Alexander Wallace: https://orcid.org/0000-0002-2894-1262

### Conflict of interest

The author declares no conflicts of interest.

## Disclaimer

Knowledge Summaries are a peer-reviewed article type which aims to answer a clinical question based on the best available current evidence. It does not override the responsibility of the practitioner. Informed decisions should be made by considering such factors as individual clinical expertise and judgement along with patient’s circumstances and owners’ values. Knowledge Summaries are a resource to help inform and any opinions expressed within the Knowledge Summaries are the author’s own and do not necessarily reflect the view of the RCVS Knowledge. Authors are responsible for the accuracy of the content. While the Editor and Publisher believe that all content herein are in accord with current recommendations and practice at the time of publication, they accept no legal responsibility for any errors or omissions, and make no warranty, express or implied, with respect to material contained within. For further information please refer to our Terms of Use.
